# Dichloridobis(pyridine-2-carboxyl­ato-κ^2^ *N*,*O*)platinum(IV) acetonitrile solvate

**DOI:** 10.1107/S1600536809017966

**Published:** 2009-05-20

**Authors:** Nam-Ho Kim, In-Chul Hwang, Kwang Ha

**Affiliations:** a School of Applied Chemical Engineering, The Research Institute of Catalysis, Chonnam National University, Gwangju 500-757, Republic of Korea; b Institute of Basic Sciences, Pohang University of Science and Technology, Pohang 790-784, Republic of Korea

## Abstract

The asymmetric unit of the title compound, [PtCl_2_(C_6_H_4_NO_2_)_2_]·CH_3_CN, contains a neutral Pt^IV^ complex and an acetonitrile solvent mol­ecule. In the complex, the Pt^4+^ atom is six-coordinated in a distorted octa­hedral environment by two N atoms and two O atoms from two pyridine­carboxyl­ate (pic) ligands and two Cl atoms. The Cl atoms are *cis* with respect to each other. The compound displays inter- and intra­molecular C—H⋯O and C—H⋯Cl hydrogen bonding.

## Related literature

For the synthesis and structure of the Pt(IV)-pic complex, [PtCl_4_(pic)]^−^, see: Griffith *et al.* (2005 ▶). For a related Pt(II)-dipicolinate complex, see: Goodgame *et al.* (1995 ▶).
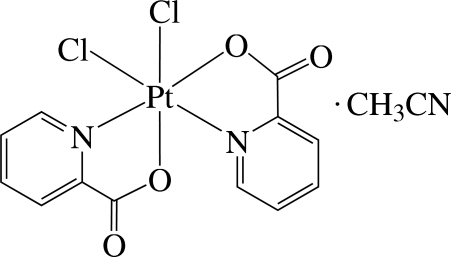


## Experimental

### 

#### Crystal data

[PtCl_2_(C_6_H_4_NO_2_)_2_]·C_2_H_3_N
*M* *_r_* = 551.25
Monoclinic, 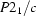

*a* = 6.103 (3) Å
*b* = 27.988 (12) Å
*c* = 9.823 (4) Å
β = 91.076 (7)°
*V* = 1677.7 (12) Å^3^
*Z* = 4
Mo *K*α radiation
μ = 8.71 mm^−1^
*T* = 293 K
0.20 × 0.15 × 0.15 mm

#### Data collection

Bruker SMART 1000 CCD diffractometer
Absorption correction: multi-scan (*SADABS*; Bruker, 2000 ▶) *T*_min_ = 0.203, *T*_max_ = 0.271
9732 measured reflections
3437 independent reflections
3051 reflections with *I* > 2σ(*I*)
*R*_int_ = 0.025

#### Refinement

*R*[*F* ^2^ > 2σ(*F* ^2^)] = 0.023
*wR*(*F* ^2^) = 0.052
*S* = 1.11
3437 reflections
218 parameters
H-atom parameters constrained
Δρ_max_ = 1.04 e Å^−3^
Δρ_min_ = −0.58 e Å^−3^

### 

Data collection: *SMART* (Bruker, 2000 ▶); cell refinement: *SAINT* (Bruker, 2000 ▶); data reduction: *SAINT*; program(s) used to solve structure: *SHELXS97* (Sheldrick, 2008 ▶); program(s) used to refine structure: *SHELXL97* (Sheldrick, 2008 ▶); molecular graphics: *ORTEP-3* (Farrugia, 1997 ▶) and *PLATON* (Spek, 2009 ▶); software used to prepare material for publication: *SHELXL97*.

## Supplementary Material

Crystal structure: contains datablocks global, I. DOI: 10.1107/S1600536809017966/bt2950sup1.cif

Structure factors: contains datablocks I. DOI: 10.1107/S1600536809017966/bt2950Isup2.hkl

Additional supplementary materials:  crystallographic information; 3D view; checkCIF report

## Figures and Tables

**Table 1:** Selected bond lengths (Å)

Pt1—O1	1.999 (3)
Pt1—N2	2.013 (3)
Pt1—O3	2.022 (3)
Pt1—N1	2.025 (4)
Pt1—Cl2	2.2910 (14)
Pt1—Cl1	2.3003 (13)

**Table 2:** Hydrogen-bond geometry (Å, °)

*D*—H⋯*A*	*D*—H	H⋯*A*	*D*⋯*A*	*D*—H⋯*A*
C2—H2⋯O2^i^	0.93	2.45	3.207 (7)	139
C7—H7⋯Cl1^ii^	0.93	2.75	3.583 (5)	150
C7—H7⋯Cl2	0.93	2.76	3.334 (5)	121
C10—H10⋯O4^iii^	0.93	2.42	3.223 (6)	145
C13—H13*A*⋯O2^iv^	0.96	2.43	3.256 (8)	144
C13—H13*B*⋯Cl1^v^	0.96	2.84	3.625 (7)	140

Symmetry codes: (i) 
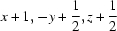
; (ii) 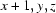; (iii) 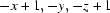; (iv) 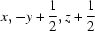; (v) 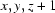.

**Fig. 1.**
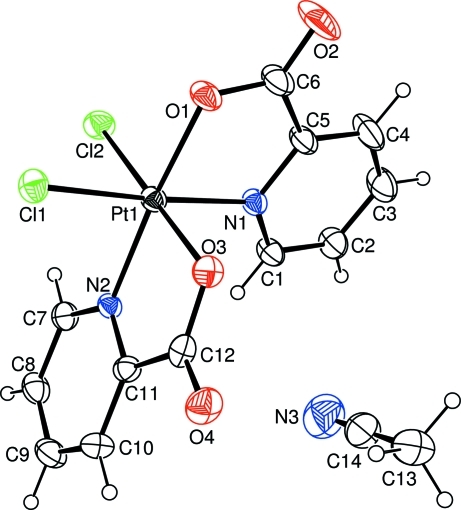
The structure of the title compound, with displacement ellipsoids drawn at the 30% probability level for non-H atoms.

**Fig. 2.**
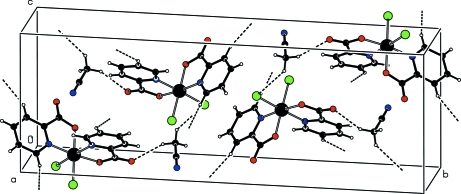
View of the unit-cell contents of the title compound. Hydrogen-bond interactions are drawn with dashed lines.

## supplementary crystallographic information

### Comment

The asymmetric unit of the title compound, [PtCl_2_(C_6_H_4_NO_2_)_2_].CH_3_CN, contains a neutral Pt^IV^ complex and a CH_3_CN solvent molecule (Fig. 1). In the complex, the Pt^4+^ ion is six-coordinated in a distorted octahedral environment by two N atoms and two O atoms from two pyridinecarboxylate (pic) anion ligands and two Cl atoms. The Cl atoms are disposed in the *cis* position. The main contributions to the distortion are the tight O—Pt—N chelate angles (82.32 (14)° and 82.16 (13)°), which result in non-linear *trans* axes (<Cl1—Pt1—N1 = 175.68 (10)°, <Cl2—Pt1—O3 = 178.67 (10)° and <O1—Pt1—N2 = 173.39 (14)°). The different *trans* effects of the Cl, O and N atoms are not distinct, because the Pt1—Cl, Pt1—O and Pt1—N bond lengths are almost equal (Pt1—Cl: 2.3003 (13) and 2.2910 (14) Å; Pt1—O 1.999 (3) and 2.022 (3) Å; Pt1—N 2.025 (4) and 2.013 (3) Å), respectively (Table 1). The compound displays inter- and intramolecular C—H···O and C—H···Cl hydrogen bonding (Table 2 and Fig. 2). There may also be weak intermolecular π-π interactions between adjacent pyridine rings, with a shortest centroid-centroid distance of 5.223 (4) Å.

### Experimental

A suspension of K_2_PtCl_6_ (0.2148 g, 0.442 mmol) and pyridine-2-carboxylic acid (0.2000 g, 1.459 mmol) in H_2_O (10 ml) was refluxed for 5 h. The formed precipitate was separated by filtration and washed with water (20 ml) and dried under vacuum, to give a pale green powder (0.2304 g). Colorless crystals suitable for X-ray analysis were obtained by slow evaporation from a CH_3_CN solution.

### Refinement

H atoms were positioned geometrically and allowed to ride on their respective parent atoms [C—H = 0.93 (aromatic) or 0.96 Å (CH_3_) and *U*_iso_(H) = 1.2*U*_eq_(C) or 1.5*U*_eq_(methyl C)].

### Crystal data

**Table:**

[PtCl_2_(C_6_H_4_NO_2_)_2_]·C_2_H_3_N	*F*(000) = 1040
*M**_r_* = 551.25	*D*_x_ = 2.182 Mg m^−^^3^
Monoclinic, *P*2_1_/*c*	Mo *K*α radiation, λ = 0.71073 Å
Hall symbol: -P 2ybc	Cell parameters from 979 reflections
*a* = 6.103 (3) Å	θ = 2.5–25.9°
*b* = 27.988 (12) Å	µ = 8.71 mm^−^^1^
*c* = 9.823 (4) Å	*T* = 293 K
β = 91.076 (7)°	Stick, colorless
*V* = 1677.7 (12) Å^3^	0.20 × 0.15 × 0.15 mm
*Z* = 4	

### Data collection

**Table:**

Bruker SMART 1000 CCD diffractometer	3437 independent reflections
Radiation source: fine-focus sealed tube	3051 reflections with *I* > 2σ(*I*)
graphite	*R*_int_ = 0.025
φ and ω scans	θ_max_ = 26.4°, θ_min_ = 1.5°
Absorption correction: multi-scan (*SADABS*; Bruker, 2000)	*h* = −6→7
*T*_min_ = 0.203, *T*_max_ = 0.271	*k* = −35→33
9732 measured reflections	*l* = −12→12

### Refinement

**Table:**

Refinement on *F*^2^	Primary atom site location: structure-invariant direct methods
Least-squares matrix: full	Secondary atom site location: difference Fourier map
*R*[*F*^2^ > 2σ(*F*^2^)] = 0.023	Hydrogen site location: inferred from neighbouring sites
*wR*(*F*^2^) = 0.052	H-atom parameters constrained
*S* = 1.11	*w* = 1/[σ^2^(*F*_o_^2^) + (0.0126*P*)^2^ + 3.1343*P*] where *P* = (*F*_o_^2^ + 2*F*_c_^2^)/3
3437 reflections	(Δ/σ)_max_ = 0.001
218 parameters	Δρ_max_ = 1.04 e Å^−^^3^
0 restraints	Δρ_min_ = −0.58 e Å^−^^3^

### Special details

**Table:**

Geometry. All e.s.d.'s (except the e.s.d. in the dihedral angle between two l.s. planes) are estimated using the full covariance matrix. The cell e.s.d.'s are taken into account individually in the estimation of e.s.d.'s in distances, angles and torsion angles; correlations between e.s.d.'s in cell parameters are only used when they are defined by crystal symmetry. An approximate (isotropic) treatment of cell e.s.d.'s is used for estimating e.s.d.'s involving l.s. planes.
Refinement. Refinement of *F*^2^ against ALL reflections. The weighted *R*-factor *wR* and goodness of fit *S* are based on *F*^2^, conventional *R*-factors *R* are based on *F*, with *F* set to zero for negative *F*^2^. The threshold expression of *F*^2^ > σ(*F*^2^) is used only for calculating *R*-factors(gt) *etc*. and is not relevant to the choice of reflections for refinement. *R*-factors based on *F*^2^ are statistically about twice as large as those based on *F*, and *R*- factors based on ALL data will be even larger.

### Fractional atomic coordinates and isotropic or equivalent isotropic displacement parameters (Å^2^)

**Table:**

	*x*	*y*	*z*	*U*_iso_*/*U*_eq_	
Pt1	0.67421 (3)	0.119200 (6)	0.082756 (17)	0.03061 (6)	
Cl1	0.47415 (19)	0.06563 (4)	−0.04645 (12)	0.0421 (3)	
Cl2	0.9275 (2)	0.12741 (4)	−0.08481 (13)	0.0454 (3)	
O1	0.5138 (5)	0.17577 (11)	0.0066 (3)	0.0444 (8)	
O2	0.4983 (9)	0.25418 (14)	0.0228 (6)	0.0971 (19)	
O3	0.4557 (5)	0.11261 (11)	0.2338 (3)	0.0389 (7)	
O4	0.3574 (6)	0.06264 (14)	0.3949 (4)	0.0533 (10)	
N1	0.8376 (6)	0.17036 (13)	0.1894 (4)	0.0336 (8)	
N2	0.8101 (6)	0.06220 (12)	0.1765 (4)	0.0297 (8)	
C1	1.0064 (8)	0.16447 (16)	0.2751 (5)	0.0410 (11)	
H1	1.0535	0.1337	0.2961	0.049*	
C2	1.1128 (9)	0.20275 (18)	0.3335 (5)	0.0504 (14)	
H2	1.2320	0.1980	0.3923	0.060*	
C3	1.0415 (10)	0.2478 (2)	0.3040 (6)	0.0610 (16)	
H3	1.1108	0.2743	0.3428	0.073*	
C4	0.8651 (11)	0.25356 (19)	0.2158 (7)	0.0680 (19)	
H4	0.8137	0.2841	0.1957	0.082*	
C5	0.7654 (9)	0.21476 (17)	0.1578 (5)	0.0452 (12)	
C6	0.5793 (10)	0.21694 (19)	0.0564 (6)	0.0550 (15)	
C7	0.9907 (8)	0.03922 (17)	0.1407 (5)	0.0380 (11)	
H7	1.0735	0.0509	0.0695	0.046*	
C8	1.0567 (9)	−0.00183 (17)	0.2081 (5)	0.0451 (12)	
H8	1.1841	−0.0176	0.1832	0.054*	
C9	0.9325 (10)	−0.01915 (18)	0.3122 (6)	0.0520 (14)	
H9	0.9745	−0.0468	0.3581	0.062*	
C10	0.7448 (9)	0.00483 (18)	0.3481 (5)	0.0476 (13)	
H10	0.6595	−0.0064	0.4187	0.057*	
C11	0.6848 (7)	0.04566 (16)	0.2781 (4)	0.0341 (10)	
C12	0.4840 (8)	0.07409 (17)	0.3081 (5)	0.0386 (11)	
N3	0.7810 (12)	0.1214 (2)	0.5485 (7)	0.091 (2)	
C13	0.4113 (12)	0.1411 (2)	0.6554 (7)	0.0761 (19)	
H13A	0.3723	0.1734	0.6331	0.114*	
H13B	0.4226	0.1376	0.7525	0.114*	
H13C	0.3007	0.1198	0.6201	0.114*	
C14	0.6175 (14)	0.1297 (2)	0.5963 (7)	0.0677 (18)	

### Atomic displacement parameters (Å^2^)

**Table:**

	*U*^11^	*U*^22^	*U*^33^	*U*^12^	*U*^13^	*U*^23^
Pt1	0.02912 (10)	0.02809 (10)	0.03449 (10)	0.00214 (7)	−0.00261 (7)	0.00113 (7)
Cl1	0.0381 (6)	0.0414 (6)	0.0465 (7)	−0.0055 (5)	−0.0040 (5)	−0.0048 (5)
Cl2	0.0479 (7)	0.0448 (7)	0.0439 (7)	−0.0037 (5)	0.0083 (5)	0.0066 (5)
O1	0.044 (2)	0.0332 (18)	0.055 (2)	0.0085 (15)	−0.0173 (17)	0.0024 (15)
O2	0.122 (4)	0.035 (2)	0.131 (4)	0.026 (2)	−0.079 (4)	−0.002 (2)
O3	0.0307 (17)	0.0435 (18)	0.0428 (19)	0.0064 (14)	0.0072 (14)	−0.0001 (15)
O4	0.048 (2)	0.063 (2)	0.049 (2)	−0.0049 (18)	0.0169 (18)	−0.0001 (18)
N1	0.033 (2)	0.030 (2)	0.037 (2)	0.0027 (16)	−0.0055 (17)	−0.0021 (16)
N2	0.031 (2)	0.0259 (18)	0.0325 (19)	0.0037 (15)	−0.0014 (16)	−0.0002 (15)
C1	0.040 (3)	0.031 (2)	0.052 (3)	0.002 (2)	−0.009 (2)	0.001 (2)
C2	0.051 (3)	0.044 (3)	0.055 (3)	−0.002 (2)	−0.019 (3)	−0.006 (2)
C3	0.069 (4)	0.039 (3)	0.074 (4)	−0.005 (3)	−0.023 (3)	−0.010 (3)
C4	0.084 (5)	0.027 (3)	0.092 (5)	0.009 (3)	−0.031 (4)	−0.009 (3)
C5	0.052 (3)	0.030 (3)	0.053 (3)	0.010 (2)	−0.013 (3)	0.000 (2)
C6	0.064 (4)	0.036 (3)	0.064 (4)	0.013 (3)	−0.024 (3)	0.001 (3)
C7	0.036 (3)	0.038 (3)	0.040 (3)	0.003 (2)	0.003 (2)	−0.002 (2)
C8	0.044 (3)	0.037 (3)	0.055 (3)	0.010 (2)	−0.007 (2)	−0.004 (2)
C9	0.065 (4)	0.034 (3)	0.057 (3)	0.004 (3)	−0.011 (3)	0.006 (2)
C10	0.060 (4)	0.042 (3)	0.041 (3)	−0.002 (3)	0.002 (3)	0.012 (2)
C11	0.036 (3)	0.035 (2)	0.032 (2)	−0.005 (2)	−0.001 (2)	0.0020 (19)
C12	0.038 (3)	0.043 (3)	0.035 (3)	−0.003 (2)	0.002 (2)	−0.009 (2)
N3	0.097 (5)	0.099 (5)	0.078 (4)	0.003 (4)	−0.014 (4)	−0.009 (4)
C13	0.097 (6)	0.065 (4)	0.067 (4)	−0.004 (4)	0.001 (4)	0.012 (3)
C14	0.092 (6)	0.057 (4)	0.053 (4)	−0.002 (4)	−0.017 (4)	−0.002 (3)

### Geometric parameters (Å, °)

**Table:**

Pt1—O1	1.999 (3)	C3—H3	0.9300
Pt1—N2	2.013 (3)	C4—C5	1.364 (7)
Pt1—O3	2.022 (3)	C4—H4	0.9300
Pt1—N1	2.025 (4)	C5—C6	1.498 (7)
Pt1—Cl2	2.2910 (14)	C7—C8	1.382 (6)
Pt1—Cl1	2.3003 (13)	C7—H7	0.9300
O1—C6	1.311 (6)	C8—C9	1.373 (7)
O2—C6	1.197 (6)	C8—H8	0.9300
O3—C12	1.312 (6)	C9—C10	1.379 (7)
O4—C12	1.205 (6)	C9—H9	0.9300
N1—C1	1.328 (6)	C10—C11	1.379 (6)
N1—C5	1.352 (6)	C10—H10	0.9300
N2—C7	1.329 (6)	C11—C12	1.495 (6)
N2—C11	1.350 (5)	N3—C14	1.134 (10)
C1—C2	1.373 (6)	C13—C14	1.432 (10)
C1—H1	0.9300	C13—H13A	0.9600
C2—C3	1.364 (7)	C13—H13B	0.9600
C2—H2	0.9300	C13—H13C	0.9600
C3—C4	1.379 (8)		
			
O1—Pt1—N2	173.39 (14)	C5—C4—H4	119.8
O1—Pt1—O3	91.24 (14)	C3—C4—H4	119.8
N2—Pt1—O3	82.16 (13)	N1—C5—C4	119.7 (5)
O1—Pt1—N1	82.32 (14)	N1—C5—C6	115.4 (4)
N2—Pt1—N1	97.41 (15)	C4—C5—C6	124.9 (5)
O3—Pt1—N1	90.57 (14)	O2—C6—O1	122.7 (5)
O1—Pt1—Cl2	89.09 (11)	O2—C6—C5	121.5 (5)
N2—Pt1—Cl2	97.51 (11)	O1—C6—C5	115.7 (4)
O3—Pt1—Cl2	178.67 (10)	N2—C7—C8	120.7 (5)
N1—Pt1—Cl2	88.19 (11)	N2—C7—H7	119.6
O1—Pt1—Cl1	93.37 (10)	C8—C7—H7	119.6
N2—Pt1—Cl1	86.89 (11)	C9—C8—C7	119.4 (5)
O3—Pt1—Cl1	89.70 (10)	C9—C8—H8	120.3
N1—Pt1—Cl1	175.68 (10)	C7—C8—H8	120.3
Cl2—Pt1—Cl1	91.57 (5)	C8—C9—C10	119.4 (5)
C6—O1—Pt1	114.4 (3)	C8—C9—H9	120.3
C12—O3—Pt1	113.7 (3)	C10—C9—H9	120.3
C1—N1—C5	120.3 (4)	C9—C10—C11	119.3 (5)
C1—N1—Pt1	127.5 (3)	C9—C10—H10	120.3
C5—N1—Pt1	112.1 (3)	C11—C10—H10	120.3
C7—N2—C11	120.9 (4)	N2—C11—C10	120.2 (4)
C7—N2—Pt1	126.8 (3)	N2—C11—C12	116.2 (4)
C11—N2—Pt1	112.1 (3)	C10—C11—C12	123.6 (4)
N1—C1—C2	121.5 (4)	O4—C12—O3	122.2 (5)
N1—C1—H1	119.2	O4—C12—C11	122.5 (5)
C2—C1—H1	119.2	O3—C12—C11	115.3 (4)
C3—C2—C1	119.2 (5)	C14—C13—H13A	109.5
C3—C2—H2	120.4	C14—C13—H13B	109.5
C1—C2—H2	120.4	H13A—C13—H13B	109.5
C2—C3—C4	118.9 (5)	C14—C13—H13C	109.5
C2—C3—H3	120.6	H13A—C13—H13C	109.5
C4—C3—H3	120.6	H13B—C13—H13C	109.5
C5—C4—C3	120.5 (5)	N3—C14—C13	178.8 (8)
			
O3—Pt1—O1—C6	89.9 (4)	C1—N1—C5—C4	−0.8 (8)
N1—Pt1—O1—C6	−0.5 (4)	Pt1—N1—C5—C4	−176.9 (5)
Cl2—Pt1—O1—C6	−88.8 (4)	C1—N1—C5—C6	177.9 (5)
Cl1—Pt1—O1—C6	179.7 (4)	Pt1—N1—C5—C6	1.8 (6)
O1—Pt1—O3—C12	173.1 (3)	C3—C4—C5—N1	1.3 (10)
N2—Pt1—O3—C12	−7.2 (3)	C3—C4—C5—C6	−177.3 (6)
N1—Pt1—O3—C12	−104.6 (3)	Pt1—O1—C6—O2	−179.1 (6)
Cl1—Pt1—O3—C12	79.7 (3)	Pt1—O1—C6—C5	1.6 (7)
O1—Pt1—N1—C1	−176.5 (4)	N1—C5—C6—O2	178.3 (6)
N2—Pt1—N1—C1	10.1 (4)	C4—C5—C6—O2	−3.0 (11)
O3—Pt1—N1—C1	92.3 (4)	N1—C5—C6—O1	−2.4 (8)
Cl2—Pt1—N1—C1	−87.2 (4)	C4—C5—C6—O1	176.3 (6)
O1—Pt1—N1—C5	−0.8 (3)	C11—N2—C7—C8	−1.0 (7)
N2—Pt1—N1—C5	−174.1 (3)	Pt1—N2—C7—C8	−174.9 (3)
O3—Pt1—N1—C5	−92.0 (4)	N2—C7—C8—C9	0.7 (7)
Cl2—Pt1—N1—C5	88.5 (3)	C7—C8—C9—C10	−0.3 (8)
O3—Pt1—N2—C7	−179.5 (4)	C8—C9—C10—C11	0.3 (8)
N1—Pt1—N2—C7	−89.9 (4)	C7—N2—C11—C10	1.0 (7)
Cl2—Pt1—N2—C7	−0.8 (4)	Pt1—N2—C11—C10	175.7 (4)
Cl1—Pt1—N2—C7	90.4 (4)	C7—N2—C11—C12	−179.2 (4)
O3—Pt1—N2—C11	6.2 (3)	Pt1—N2—C11—C12	−4.5 (5)
N1—Pt1—N2—C11	95.7 (3)	C9—C10—C11—N2	−0.7 (7)
Cl2—Pt1—N2—C11	−175.1 (3)	C9—C10—C11—C12	179.6 (5)
Cl1—Pt1—N2—C11	−84.0 (3)	Pt1—O3—C12—O4	−173.6 (4)
C5—N1—C1—C2	−0.3 (8)	Pt1—O3—C12—C11	6.7 (5)
Pt1—N1—C1—C2	175.1 (4)	N2—C11—C12—O4	178.8 (4)
N1—C1—C2—C3	0.9 (8)	C10—C11—C12—O4	−1.4 (7)
C1—C2—C3—C4	−0.3 (9)	N2—C11—C12—O3	−1.5 (6)
C2—C3—C4—C5	−0.8 (10)	C10—C11—C12—O3	178.3 (4)

### Hydrogen-bond geometry (Å, °)

**Table:**

*D*—H···*A*	*D*—H	H···*A*	*D*···*A*	*D*—H···*A*
C2—H2···O2^i^	0.93	2.45	3.207 (7)	139
C7—H7···Cl1^ii^	0.93	2.75	3.583 (5)	150
C7—H7···Cl2	0.93	2.76	3.334 (5)	121
C10—H10···O4^iii^	0.93	2.42	3.223 (6)	145
C13—H13A···O2^iv^	0.96	2.43	3.256 (8)	144
C13—H13B···Cl1^v^	0.96	2.84	3.625 (7)	140

Symmetry codes: (i) *x*+1, −*y*+1/2, *z*+1/2; (ii) *x*+1, *y*, *z*; (iii) −*x*+1, −*y*, −*z*+1; (iv) *x*, −*y*+1/2, *z*+1/2; (v) *x*, *y*, *z*+1.
